# Thrombopoietin Receptor Levels in Tumor Cell Lines and Primary Tumors

**DOI:** 10.1155/2010/135354

**Published:** 2010-12-28

**Authors:** Connie L. Erickson-Miller, Antony Chadderton, Anna Gibbard, Jennifer Kirchner, Kodandaram Pillarisetti, Katherine Baker, Lini Pandite, Iman El-Hariry, Yasser Mostafa Kamel, Yuan Liu, Anne-Marie Martin, Conrad Messam

**Affiliations:** ^1^Oncology Biology, GlaxoSmithKline, 1250 South Collegeville Road, Collegeville, PA 19426-2990, USA; ^2^Oncology Clinical, GlaxoSmithKline, 5 Moore Drive, Research Triangle Park, NC 27709, USA; ^3^Oncology Biomarkers, GlaxoSmithKline, Astellas Pharmaceuticals, 3 Parkway North, Deerfield, IL 60015, USA; ^4^Oncology Clinical, GlaxoSmithKline, Oncology R&D, Building 11, 1-3 Bridge Road, Stockley Park West, Uxbridge, Middlesex UB11 1BT, UK

## Abstract

Thrombopoietin (TPO) receptor agonists represent a new approach for the treatment of thrombocytopenia, which may develop as a consequence of immune thrombocytopenia, chemotherapy treatment, chronic hepatitis C infection, or myelodysplastic syndromes. There are concerns that use of certain growth factors can hasten disease progression in some types of hematologic malignancies and solid tumors. In this study, expression of *MPL* (TPO-R) mRNA was examined in tumor cell lines, patient tumor samples (renal cell carcinoma, prostatic carcinoma, soft tissue and bony/cartilage sarcoma, colon cancer, and lymphoma), and normal tissues using microarray analysis and qRT-PCR. *MPL* mRNA is expressed at very low or undetectable levels compared with erythropoietin receptor (*EPOR*), human epidermal growth factor (*ERBB2; HER2*), and insulin-like growth factor-1 receptor (*IGF1R*) in these patient samples. These data suggest TPO-R agonists will likely preferentially stimulate proliferation and differentiation of cells of megakaryocytic lineage, potentially demonstrating their utility for correcting thrombocytopenia in clinical settings.

## 1. Introduction

Thrombopoietin (TPO) is the key cytokine involved in the regulation of thrombopoiesis. Its activities are twofold: TPO stimulates both megakaryocyte colony formation and enhances megakaryocyte maturation. Megakaryocytes and their precursors possess the thrombopoietin receptor (TPO-R; *MPL*) on their surfaces. TPO is constitutively expressed, but the level of circulating TPO is directly related to platelet mass [1–5]. The JAK-STAT signal transduction pathway is activated upon binding of TPO to its receptor, resulting in changes in gene expression that promote megakaryocytic differentiation, increase megakaryocyte ploidy, and stimulate the release of platelets into the peripheral circulation. 

Thrombocytopenia, defined as a platelet count less than 150,000/*μ*L, represents an important clinical issue because it can increase the risk for bleeding. Spontaneous bleeding can occur when platelet counts drop below 20,000/*μ*L [6]. It develops as a result of an imbalance between platelet production and platelet consumption. Thrombocytopenia develops commonly in patients with a variety of conditions including HIV, chronic hepatitis C infection, portal hypertension, systemic lupus erythematosus, myelodysplastic syndromes, and cancer [7–11]. Thrombocytopenia can also result from myelosuppressive chemotherapy, although bleeding episodes are infrequent overall. Thrombocytopenia can result in dose delays and dose reductions of chemotherapy that could negatively impact disease control [11]. However, bleeding is associated with poor performance status, multiple comorbidities, bone marrow metastases, and low baseline platelet counts, and, when it occurs, bleeding predicts poor clinical outcomes [11]. Although development of recombinant TPO was discontinued because of neutralizing antibody formation, 2 TPO-R agonists, eltrombopag and romiplostim, have been approved for the treatment of chronic immune thrombocytopenic purpura (ITP) in the United States. These TPO-R agonists stimulate TPO-dependent cell lines via JAK-STAT signaling and demonstrate increased platelets in humans [6, 12–15].

Other growth factors have the potential to stimulate growth of tumors. A recent review article described the use of erythropoiesis-stimulating agents to increase hemoglobin levels in patients with cancer; numerous studies were identified in which use of these drugs increased the risk of treatment-associated death, perhaps through tumor progression [16, 17]. Erythropoietin receptor messenger ribonucleic acid (mRNA) and/or protein has been identified in a wide range of cancers, leading to the speculation that tumor progression may be a consequence of activation of endogenous erythropoietin receptors by exogenous erythropoiesis-stimulating agents, thereby promoting tumor cell proliferation and angiogenesis and inhibiting apoptosis. Acute myelogenous leukemia (AML) blast cells express *MPL* mRNA and protein [18]. TPO stimulated blast colony formation in samples from approximately 50% of patients with AML in one study [19], and enhanced proliferation of a megakaryocytic leukemic cell line [20]. Results from other investigators confirmed this and demonstrated that TPO induced cell cycle activation and could protect AML blasts from programmed cell death [21]. Eltrombopag does not appear to stimulate proliferation of AML/myelodysplastic syndromes (MDSs) patient blood and marrow samples or nonmegakaryocytic leukemia and lymphoma cell lines [22–24]. In fact, there is a decrease in proliferation at physiologically achievable concentrations of eltrombopag. Although the intensity of signaling induced by eltrombopag is lower than that induced by TPO, a mechanism for this differential effect has not been identified [14, 22, 23].

There is little quantitative information available on the expression of TPO-R on solid tumors. In a recent examination of breast, nonsmall cell lung cancer (NSCLC), and ovarian tumor samples, very little, or more often, no *MPL* expression was detectable on these patient samples [25]. To further our knowledge of *MPL* mRNA expression, quantitative reverse transcription-polymerase chain reaction (qRT-PCR) and microarray analysis were performed on tumor cell lines and in tumor samples from renal cell carcinomas, prostate cancers, lymphomas, colon cancers, and sarcomas.

## 2. Methods

### 2.1. Tumor Cell Lines

Three hundred and fifty-five cell lines of various tissue types were obtained from the American Type Culture Collection (Rockville, MD, USA), the German Collection of Microorganisms and Cell Cultures (Braunschweig, Germany), the National Cancer Institute (Bethesda, MD, USA), and the European Collection of Cell Cultures (Salisbury, UK). All cells were in log phase growth at the time of collection. The complete list of tumor cell lines used in this study is provided in the Supplementary Material available online at doi: 10.1155/2010/135354.

### 2.2. Patient Tissue Samples

Colon (5 normal, 42 tumor), prostate (7 normal, 30 tumor, 11 benign hypertrophy), and lymphoma (3 normal spleen, 3 normal lymph nodes, 42 lymphoma) cDNA samples were obtained from Cytomyx LLC (Lexington, MA, USA). Archival tissue samples were also obtained from 105 patients with locally advanced or metastatic renal cell carcinoma (RCC) who failed, or who were unable to tolerate, first-line cytokine-based therapy. These patients were selected for epidermal growth factor receptor (EGFR) and/or *ERBB2* positive (1+, 2+, or 3+) tumors. Archival tissue samples were obtained from an additional 46 patients who had metastatic or locally recurrent RCC of clear cell histology. These individuals either had no prior systemic therapy or had failed only 1 prior cytokine-based or bevacizumab-based therapy.

Additional archival tissue samples from 22 patients with soft tissue sarcomas (14 leiomyosarcomas, 8 malignant fibrous histiocytomas) and 12 bony/cartilage sarcomas (3 chondroblastomas, 7 osteosarcomas, 1 chordoma, 1 enchondroma) were obtained from the University of Pennsylvania (Philadelphia, PA, USA).

### 2.3. Microarray Analysis

Clinical tissue samples were all formalin fixed and paraffin embedded. RNA is often fragmented in these samples. To ensure the quality of RNA extraction, tumor tissues were microdissected using laser capture microdissection. RNA was rapidly extracted; 100 base pair (bp), 300 bp, and 400 bp segments of the *β*-actin gene were tested using qRT-PCR. Samples with less than 32 cycle threshold (Ct) at the 300 bp level and less than 34 Ct at the 400 bp level were included in the microarray analysis.

Microarray analysis of all RCC samples was performed at Response Genetics, Inc. (Los Angeles, CA, USA) using the Affymetrix HG-U133 Plus2 chip. Microarray analysis was performed on the sarcoma tissue samples, using the Affymetrix HG-U133 chip, at the University of Pennsylvania. RNA microarray gene signal intensity was normalized using robust microarray average (RMA) analysis as described by Irizarry [26].

### 2.4. qRT-PCR Analysis

Primers and probes were customly made by Integrated DNA Technologies (Coralville, IA, USA). Sequences for the primers and probes used in this study are listed in the Supplementary material. 

Cells from each cell line were lysed in Trizol reagent (Invitrogen). After phase separation with chloroform, total RNA was extracted using the RNeasy Mini Kit (Qiagen, Germantown, MD, USA) following the manufacturer's instructions. Genomic DNA contamination was removed using DNase I (Ambion, Austin, TX, USA). RNA samples were considered to be free of genomic DNA if no amplification was observed in a standard TaqMan assay using 10 ng of RNA and ACTB primer/probe oligonucleotides. The RNA was quantified using a Nanodrop analyzer and was subsequently converted to cDNA by reverse transcription utilizing the High Capacity cDNA Archive Kit (Applied Biosystems Inc., Foster City, CA, USA). 

qRT-PCR was also performed on the cDNA from both the normal and tumor tissue samples and from the cell lines to assess the levels of *MPL*, erythropoietin receptor (*EPOR*), human epidermal growth factor (*ERBB2*; *HER2*), and insulin-like growth factor-1 receptor (*IGF1R*). Data were normalized to a set of housekeeping genes used as internal standards including *GADPH*, *PPIA* (cyclophilin-A), and *ACTB* (*β*-actin). The equivalent of 10 ng mRNA per well was arrayed into 384-well plates using a Biomek FX robot (Beckman Coulter, Inc., Fullerton, CA, USA) and qRT-PCR was carried out using a 7900HT Sequence Detector System (Applied Biosystems Inc.) in a 5 *μ*L reaction volume. TaqMan Universal PCR Master Mix 2X (Applied Biosystems Inc.) and universal PCR conditions recommended by the manufacturer were followed. 

Ct values were analyzed using in-house analysis software in which values were normalized to the internal housekeeping genes in the reaction. Only samples with sufficient housekeeping gene expression (Ct < 32) were used. Abundance was calculated using the formula: abundance = 10e((40 − Ct)/3.35). To normalize the data, samples were scaled relative to each other using the geometric mean of the set of valid housekeeping gene datapoints for that sample. Each datapoint was then expressed as the ratio of the housekeeping gene abundance in the sample to the average abundance of that housekeeping gene in all samples and marked invalid if it had statistically inconsistent behavior with the other housekeeping genes in those samples with similar tissue types.

## 3. Results

**Figure 1:**
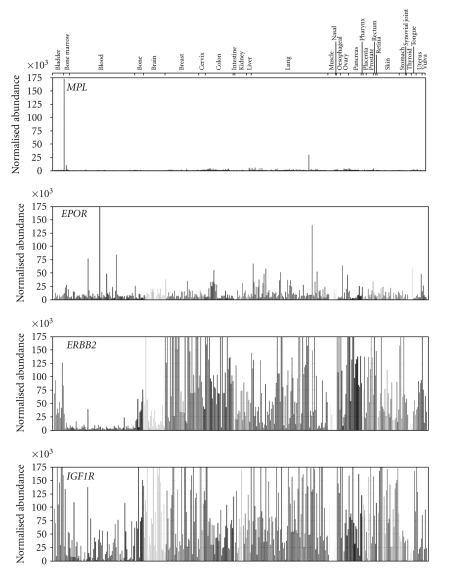
Quantitative reverse transcription-polymerase chain reaction measurement of *MPL*, *EPOR*, *ERBB2*, and *IGF1R* mRNA in various tumor cell lines. Data were normalized to a set of housekeeping genes (GAPDH, cyclophilin, and *β*-actin). The normalized abundance of mRNA (*y*-axis) is scaled to 175,000 for all samples. Cycle threshold (Ct) values were calculated using the following equation: Abundance = 10e((40 − Ct)/3.35). Samples were scaled relative to each other using the geometric mean of the set of valid housekeeping gene datapoints for that sample.

In most of the 355 tumor cell lines analyzed, *MPL* mRNA was expressed only at very low levels (Figure 1). Only 3 cell lines demonstrated expression of *MPL* mRNA in excess of 9500 normalized abundance, as illustrated in Figure 1. These were the erythroleukemia cell lines HEL 92.1.7 and KG-1 and the lung tumor cell line NCI-H510.

Table 1 summarizes normalized mRNA abundance for *MPL*, *EPOR*, *ERBB2*, and *IGF1R* in all the cell lines studied. *MPL* expression was minimal. The mean normalized abundance for *MPL* mRNA was 1447 and the mode was 621. *EPOR* mRNA, in contrast, was expressed at low to moderate levels (mean 12,587; mode 7811) and *ERBB2* mRNA expression was markedly greater (mean 280,190; mode 40,828) as was *IGF1R* mRNA expression mean 78,977; mode 56,625). The highest expression of *ERBB2* was observed in breast tumor cell lines, as expected. *ERBB2* and *IGF1R* mRNA demonstrated particularly higher expression than *MPL* or *EPOR* in lung, brain, breast, and bladder cell lines, as shown in Figure 1. 

**Figure 2:**
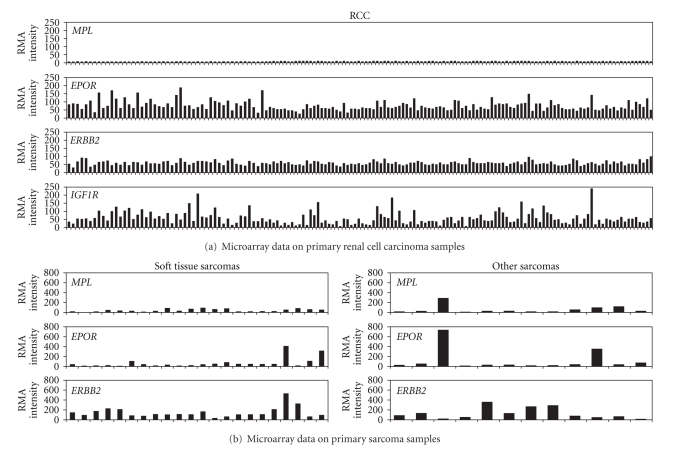
Measurement of *MPL*, *EPOR*, *ERBB2*, and *IGF1R* mRNA in renal cell carcinoma and soft tissue and bony/cartilage sarcoma tumor samples using microarray analysis. Robust microarray average (RMA) was used for assay normalization. The levels of gene expression of *MPL*, *EPOR*, *ERBB2*, and *IGF1R* mRNA were reported as RMA intensity values.

Microarray data analysis of *MPL* mRNA expression was performed on primary tumor samples from RCC and soft tissue and bony/cartilage sarcomas. None of the 151 RCC tumors expressed *MPL* mRNA at detectable levels (RMA ≥ 50) (Figure 2, Table 2). However, 132 (87%) showed detectable levels of *EPOR* mRNA, and 122 (81%) demonstrated *ERBB2* mRNA expression. Fewer RCC tumor samples (81; 54%) expressed detectable *IGF1R* mRNA. *EPOR*, *ERBB2*, and *IGF1R* mRNA levels were overall lower in these RCC samples than those observed in the breast and NSCLC tumors previously described [25].

Seven of 22 (32%) soft tissue sarcomas and 4/12 bony/cartilage sarcomas (33%) expressed *MPL *mRNA at detectable levels (Figure 2(b), Table 2). Similarly, 6/22 (27%) soft tissue and 4/12 (33%) bony/cartilage samples showed detectable levels of *EPOR*. The majority of samples (21/22 (95%) and 9/12 (75%), respectively) expressed detectable *ERBB2* mRNA.

Microarray analysis is best used to describe relative differences in mRNA levels. qRT-PCR analysis was performed on additional samples to more quantitatively determine mRNA expression in normal tissues and tumor samples. The qRT-PCR data were normalized to a set of 3 housekeeping genes. 

**Figure 3:**
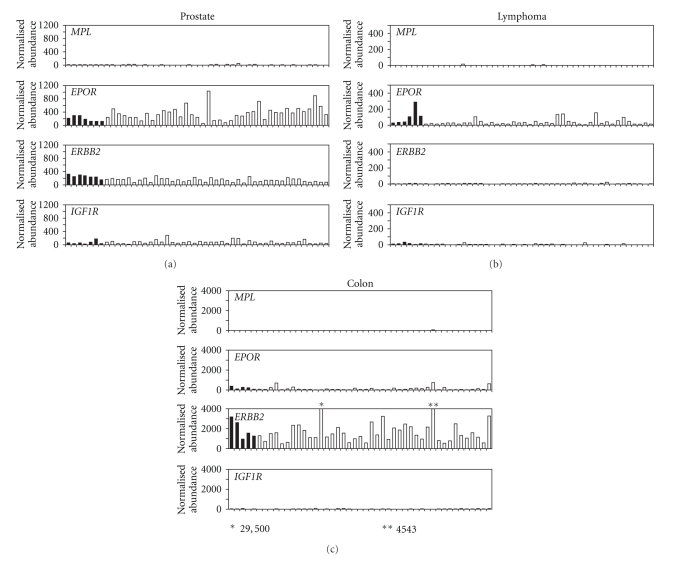
Quantitative reverse transcription-polymerase chain reaction measurement of *MPL*, *EPOR*, *ERBB2*, and *IGF1R* mRNA in various normal tissue samples and tumor samples. Data were normalized to a set of housekeeping genes (*GAPDH*, *PPIA*, and *ACTB*). Cycle threshold (Ct) values were calculated using the following equation: Abundance = 10e((40 − Ct)/3.35). Samples were scaled relative to each other using the geometric mean of the set of valid housekeeping gene datapoints for that sample.

Among prostate samples, no normal or benign prostatic hypertrophy samples expressed detectable *MPL* mRNA whereas 1 tumor (3%) expressed *MPL *(Table 3, Figure 3). All samples expressed detectable levels of *EPOR* and *ERBB2*. *IGF1R *mRNA expression was also common with 4/7 (57%) normal prostate samples, 25/30 (83%) prostate tumors, and 7/11 (64%) benign prostatic hypertrophy samples expressing detectable levels.

None of the 6 normal lymph node and spleen samples or 42 lymphoma samples expressed detectable levels of *MPL*, *ERBB2*, or *IGF1R* mRNA (Table 3, Figure 3). In contrast, 3/6 (50%) normal lymph node and spleen samples and 10/42 (24%) lymphoma samples expressed detectable *EPOR* mRNA. 

None of the 5 normal colon samples or 42 colon tumors expressed detectable *MPL* mRNA (Table 3, Figure 3). *IGF1R *mRNA expression was detectable in 1 (20%) normal and 5 (12%) adenocarcinoma of the colon samples. However, all of the normal colon samples and 27 (64%) colon tumors expressed detectable *EPOR *mRNA and all samples expressed detectable *ERBB2*.

The stage of disease was available for the samples of lymphoma and colon and prostate tumors, however, due to the lack of detectable expression of *MPL* mRNA in these tumor types, no interpretation could be made on the relationships between expression levels and stage of disease.

## 4. Discussion

Thrombocytopenia can be a consequence of diminished platelet production in the bone marrow, or increased platelet consumption in the spleen, liver, or circulation. Currently, platelet transfusions are used for the treatment of acute thrombocytopenia due to a variety of etiologies [27]. However, significant limitations exist to the use of platelet transfusions. Many patients become refractory to transfusion. Limited availability and cost concerns further impact the utility of platelet transfusion as an optimal treatment for thrombocytopenia. Despite donor testing and pathogen inactivation systems, infections with HIV, hepatitis B or C viruses, cytomegalovirus, or bacteria are possible. Alloimmunization and febrile transfusion reactions are 2 of the immunologically mediated adverse events that can follow platelet transfusion. And, transfusion-related acute lung injury is a potentially fatal immunologic consequence of transfusions of plasma containing blood products. Therefore, alternative treatments with fewer inherent challenges are sought. TPO-R agonists represent a class of such agents. Eltrombopag is approved for use in patients with chronic ITP and is currently in clinical trials for thrombocytopenia in patients with MDS/secondary AML and for thrombocytopenia due to chemotherapy in patients with solid tumors. 

The expression of *EPOR*, *ERBB2,* and *IGF1R* genes in tumors has been well studied and is widely reported, thus the expression of these genes was used as a comparator for *MPL *[28–33].

It should be understood that expression of these genes does not always mean that the protein is detected on the cell surface. Nor does expression of a receptor protein necessarily reflect signaling or proliferation can occur [34, 35]. The expression of TPO-R protein by Western blotting of several lung tumor cell lines, including NCI-H510, which was one of the 3 cell lines that demonstrated high mRNA expression in this study, showed no protein expression in any of these lines [25]. Although, there are reports of AML cells-expressing *MPL*, 2 recent studies found no proliferation of cells in blood and marrow samples from patients with AML or MDS/secondary AML [22], or in leukemia and lymphoma cell lines in response to eltrombopag [23]; in fact, there is a decrease in proliferation at physiologically achievable concentrations of eltrombopag [22, 23].

Activity of TPO-R agonists depends on the expression of TPO-R in the tissue of interest. Likewise, expression of TPO-R on cells other than megakaryocytes is of great interest; activation of growth factor receptors such as TPO-R could theoretically enhance proliferation in such tissue. The goal of this study, therefore, was to expand on the *MPL* mRNA expression data by microarray and qRT-PCR previously described for breast, lung, and ovarian tumors [25] into other tumor types and tumor cell lines. *MPL *mRNA was consistently expressed at low or undetectable levels in the 355 tumor cell lines studied. *MPL* mRNA was expressed at detectable levels in 3 cell lines, the erythroleukemia lines HEL92.1.7 and KG-1, and the NCI-H510 lung tumor cell line. The HEL92.1.7 line serves as a positive control cell line in this study, as it expresses some MPL mRNA and gives a very slight, but statistically significant, proliferative response to eltrombopag [23]. A comparison of the *MPL* mRNA expression in this line to several other leukemia cell lines, including the NOMO-1 F36-P and ML-2 cell lines, also represented in Figure 1, was previously reported [23]. In comparison, *EPOR* mRNA was expressed in low-to-moderate levels and *ERBB2* and *IGF1R* mRNA were expressed at higher levels. As expected, breast tumor cell lines typically expressed much higher levels of *ERBB2* mRNA. This tumor cell line data reinforced data in samples of breast and lung tumor from patients [25].

Microarray data of tumor samples revealed similar results. *MPL* mRNA levels were low or below the limits of detection in all tumor samples examined. The majority of tumor samples expressed mRNA for *EPOR*, *ERBB2*, and *IGF1R* at varying levels. One caveat to this type of data is that the expression on normal tissues of each type are not measured. To this end, qRT-PCR of both normal tissues and tumor samples permitted a direct comparison of mRNA levels. *MPL* mRNA expression was low in both normal tissues and in prostate, colon, and lymphoma tumor samples, and *MPL* mRNA was not increased in any tumor tissue relative to normal tissue.

## 5. Conclusions

Because the levels of *MPL *mRNA are low or undetectable in RCC, sarcoma, prostate, and colon tumors as well as lymphoma samples, it is unlikely that TPO-R agonists can induce proliferation of these types of tumor cells. Rather, TPO-R agonists will likely preferentially stimulate proliferation and differentiation of megakaryocytic cells to produce platelets and relieve thrombocytopenia. These data provide additional support for continued study of TPO-R agonists for the treatment of oncology-related thrombocytopenia and demonstrate their potential utility for correcting thrombocytopenia in clinical settings.

##  Conflict of Interests

C. Erickson-Miller, Y. Liu, C. Messam, K. Baker, Y. Mostafa Kamel, L. Pandite, A.-M. Martin, K. Pillarisetti, A. Chadderton, and J. Kirchner are employees of GlaxoSmithKline. As employees of GlaxoSmithKline, C. Erickson-Miller, Y. Liu, C. Messam, K. Baker, Y. Mostafa Kamel, L. Pandite, and A.-M. Martin own GlaxoSmithKline stock. A. Gibbard is an employee of Morphotek. I. El-Hariry is currently employed by Astellas Pharmaceuticals, and is a former employee of GlaxoSmithKline who took part in creation of this paper.

## Supplementary Material

The supplemental data section contains a list of the cell lines (in order of appearance in Figure 1), their original tumor type and the source, for the data in Figure 1. The supplemental data section also contains the sequences for the primers and probes for MPL, EPOR, ERBB2, IGF1R, GAPDH, PPIA and ACTB used in the qRT-PCR experiments described in this paper.

## Figures and Tables

**Table 1:** Normalized Abundance of *MPL*, *EPOR*, *ERBB2*, and *IGF1R* mRNA Across 355 Tumor Cell Line Types.

	Cycle Threshold Values				
	Mode	Mean	SD	Minimum	Maximum
*MPL*	621	1447	463	1	172,402
*EPOR*	7811	12,587	1055	1	311,201
*ERBB2*	40,828	280,190	63,578	197	12,632,200
*IGF1R*	56,625	78,977	3914	1	535,859

*EPOR*: erythropoietin receptor; *ERBB2*: human epidermal growth factor; *IGF1R*: insulin growth factor-1 receptor; *MPL*: thrombopoietin receptor; SD: standard deviation.

Cycle threshold values were calculated and samples normalized as described in the Materials and Methods section.

**Table 2:** Number (%) of Primary Tumor Samples With Detectable *MPL* mRNA Expression Determined by Microarray Analysis.

*n* (%)	RCC *n* = 151	Soft Tissue Sarcomas *n* = 22	Bony/Cartilage Sarcomas *n* = 12
*MPL*	0	7 (32)	4 (33)
*EPOR*	132 (87)	6 (27)	4 (33)
*ERBB2*	122 (81)	21 (95)	9 (75)
*IGF1R*	81 (54)	ND	ND

*EPOR*: erythropoietin receptor; *ERBB2*: human epidermal growth factor; *IGF1R*: insulin growth factor-1 receptor; *MPL*: thrombopoietin receptor; ND: not determined; RCC: renal cell carcinoma; RMA: robust microarray averages. Detectable expression is determined by a RMA ≥ 50.

**Table 3:** Number (%) of Primary Tumor Samples With Detectable mRNA Expression Determined by qRT-PCR.

*n* (%)	Prostate Normal *n* = 7	Prostate Tumor *n* = 30	Benign Prostatic Hypertrophy *n* = 11	Normal Lymph Node and Spleen *n* = 6	Lymphoma *n* = 42	Colon Normal *n* = 5	Colon Tumor *n* = 42
*MPL*	0	1 (3)	0	0	0	0	0
*EPOR*	7 (100)	30 (100)	11 (100)	3 (50)	10 (24)	5 (100)	27 (64)
*ERBB2*	7 (100)	30 (100)	11 (100)	0	0	5 (100)	42 (100)
*IGF1R*	4 (57)	25 (83)	7 (64)	0	0	1 (20)	5 (12)

*EPOR*: erythropoietin receptor; *ERBB2*: human epidermal growth factor; *IGF1R*: insulin growth factor-1 receptor; *MPL*: thrombopoietin receptor; qRT-PCR: quantitative reverse transcription-polymerase chain reaction.

Detectable expression is determined by a normalized abundance ≥50.

## Abbreviations

AML: Acute myelogenous leukemia
ATCC: American Type Culture Collection
Ct: Cycle threshold
DSMZ: German Collection of Microorganisms and Cell Cultures
ECACC: European Collection of Cell Cultures
EGFR: Epidermal growth factor receptor
*EPOR*: Erythropoietin receptor
*ERBB2*: Human epidermal growth factor
ITP: Immune thrombocytopenic purpura
*IGF1R*: Insulin-like growth factor-1 receptor
MDS: Myelodysplastic syndromes
mRNA: Messenger ribonucleic acid
NCI: National Cancer Institute
ND: Not determined
NSCLC: Nonsmall cell lung cancer
qRT-PCR: Quantitative reverse transcription-polymerase chain reaction
RCC: Renal cell carcinoma
RMA: Robust microarray average
SD: Standard deviation
TPO: Thrombopoietin
TPO-R: Thrombopoietin receptor.
