# Supplementary material for: Thrombopoietin Receptor Levels in Tumor Cell Lines and Primary Tumors
Source: J Oncol. 2010 Dec 28;2010:135354. doi: 10.1155/2010/135354 (PMC3026977; doi:10.1155/2010/135354)
Supplement: Supplementary file 1 — The supplemental data section contains a list of the cell lines (in order of appearance in Figure 1), their original tumor type and the source, for the data in Figure 1. The supplemental data section also contains the sequences for the primers and probes for MPL, EPOR, ERBB2, IGF1R, GAPDH, PPIA and ACTB used in the qRT-PCR experiments described in this paper. [file 135354.f1.pdf]

Supplemental Data

Primers used in the TPO-R expression study.

MPL	
Forward	AGTGGAACCCAGCCTCCTTG
Reverse	CTGCAATCTTCGGTAGTCCATCTG
Probe	FAM-CAAGTCCTCAGAGGACTCCTTTGCCC-TAMRA
EPOR	
Forward	GCTATGTGGCTTGCTCTTAGGACAC
Reverse	CCATCCCTGTTCCATAAGTCTTGAG
Probe	FAM-ATTGGATCCCTGATCATCTGCAGCCTG
ERBB2	
Forward	AGCCCAGCCTTCGACAACC
Reverse	GTCCCTTTGAAGGTGCTGGG
Probe	FAM-ATTACTGGGACCAGGACCCACCAGAGC
IGF1R	
Forward	GATTCAGATGGCCGGAGAGA
Reverse	TGTGGACGAACTTATTGGCG
Probe	FAM-TGCAGACGGCATGGCATACCTCA
GAPDH	
Forward	CAAGGTCATCCATGACAACTTTG
Reverse	GGGCCATCCACAGTCTTCTG
Probe	FAM-ACCACAGTCCATGCCATCACTGCC A-TAMRA
PPIA	
Forward	CATCTGCACTGCCAAGACTGA
Reverse	TGCCTTCTTTCACTTTGCCA
Probe	FAM-CACCACATGCTTGCCATCCAACCA-TAMRA
ACTB	
Forward	GAGCTACGAGCTGCCTGACG
Reverse	GATGTTTCGTGGATGCCACAGGAC
Probe	FAM-CATCACCATTGGCAATGAGCGGTT CC-TAMRA

Cell lines used in this study. Human tumor cell line name, tissue of origin and donor description used for quantitative PCR analysis. Cell lines are listed in the order presented in the graphs.

Cell Line	Tissue	Tumor Type	Cell Source
NCI-H295R	Adrenal gland	Adrenal gland carcinoma	ATCC
5637	Bladder	Carcinoma	ATCC
639-V	Bladder	Transitional cell carcinoma	DSMZ
647-V	Bladder	Carcinoma	DSMZ
BFTC-905	Bladder	Transitional cell carcinoma	DSMZ
HT-1197	Bladder	Transitional cell carcinoma	ATCC
HT1376	Bladder	Transitional cell carcinoma	ATCC
J82	Bladder	Transitional cell carcinoma	ATCC
SCaBER	Bladder	Squamous cell carcinoma	ATCC
SW780	Bladder	Transitional cell carcinoma	ATCC
T24	Bladder	Transitional cell carcinoma	DSMZ
UM-UC-3	Bladder	Transitional cell carcinoma	ATCC
1A2	Blood/marrow	B lymphoblast leukemia	ATCC
ARH-77	Blood/marrow	Plasma cell myeloma	ATCC
BC-1	Blood/marrow	B-cell lymphoma	ATCC
BC-2	Blood/marrow	B-cell lymphoma	ATCC
BC-3	Blood/marrow	B-cell lymphoma	ATCC
BDCM	Blood/marrow	Acute myeloid leukemia	ATCC
BV-173	Blood/marrow	Chronic myeloid leukemia	DSMZ
CA46	Blood/marrow	Burkitt's lymphoma	ATCC
CCRF-CEM	Blood/marrow	Acute lymphoblastic leukemia	ATCC
CEM/C1	Blood/marrow	Acute lymphoblastic leukemia	ATCC
CESS	Blood/marrow	Acute myeloid leukemia	ATCC
CRO-AP2	Blood/marrow	B-cell lymphoma	DSMZ
CRO-AP5	Blood/marrow	B-cell lymphoma	DSMZ

Cell Line	Tissue	Tumor Type	Cell Source
Daudi	Blood/marrow	Burkitt's lymphoma	ATCC
DB	Blood/marrow	Diffuse large B cell lymphoma	ATCC
DG-75	Blood/marrow	Burkitt's lymphoma	ATCC
EB-1	Blood/marrow	Burkitt's lymphoma	DSMZ
EB-2	Blood/marrow	Burkitt's lymphoma	ATCC
EB-3	Blood/marrow	Burkitt's lymphoma	ATCC
F-36P	Blood/marrow	Acute myeloid leukemia	DSMZ
HD-MY-Z	Blood/marrow	Hodgkin disease	DSMZ
HEL 92.1.7	Blood/marrow	Erythroleukemia	ATCC
HH	Blood/marrow	Cutaneous T-cell lymphoma	ATCC
HL-60	Blood/marrow	Acute myeloid leukemia	DSMZ
HS-Sultan	Blood/marrow	Burkitt's lymphoma	ATCC
HT	Blood/marrow	B-cell lymphoma	DSMZ
HuT-78	Blood/marrow	Cutaneous T-cell lymphoma	ATCC
J.RT3-T3.5	Blood/marrow	Acute lymphoblastic leukemia	ATCC
Jiyoye	Blood/marrow	Burkitt's lymphoma	ATCC
Jurkat	Blood/marrow	Acute lymphoblastic leukemia	ATCC
JVM-3	Blood/marrow	Acute B-cell lymphocytic leukemia	DSMZ
K-562	Blood/marrow	Chronic myeloid leukemia	ATCC
Kasumi-2	Blood/marrow	B-cell leukemia	DSMZ
KG-1	Blood/marrow	Acute myeloid leukemia	ATCC
KU812	Blood/marrow	Chronic myeloid leukemia	DSMZ
L-428	Blood/marrow	Hodgkin disease	DSMZ
MC/CAR	Blood/marrow	Plasmacytoma/myeloma	ATCC
MC-116	Blood/marrow	B-cell lymphoma	DSMZ
MEC-1	Blood/marrow	B cell lymphocytic leukemia	DSMZ

Cell Line	Tissue	Tumor Type	Cell Source
MEG-01	Blood/marrow	Chronic myeloid leukemia	ATCC
MHH-PREB-1	Blood/marrow	B-cell lymphoma	DSMZ
MJ	Blood/marrow	Cutaneous T-cell lymphoma	ATCC
ML-2	Blood/marrow	Acute myeloid leukemia	DSMZ
MOLT-16	Blood/marrow	Acute lymphoblastic leukemia	DSMZ
MOLT-4	Blood/marrow	Acute lymphoblastic leukemia	ATCC
MV-4-11	Blood/marrow	Biphenotypic B-cell myelomonocytic leukemia	ATCC
NALM-6	Blood/marrow	Acute B-cell lymphocytic leukemia	DSMZ
NAMALWA	Blood/marrow	Burkitt's lymphoma	ATCC
NOMO-1	Blood/marrow	Acute myeloid leukemia	DSMZ
OCI-M1	Blood/marrow	Acute myelogenous leukemia	DSMZ
P3HR1	Blood/marrow	Burkitt’s lymphoma	ATCC
PLB-985	Blood/marrow	Promyelocytic leukemia	DSMZ
RAJI	Blood/marrow	Burkitt's lymphoma	ATCC
RCH-ACV	Blood/marrow	Acute lymphoblastic leukemia	DSMZ
RC-K8	Blood/marrow	B-cell lymphoma	DSMZ
REC-1	Blood/marrow	Diffuse non-Hodgkin lymphoma	ATCC
RL	Blood/marrow	Non-Hodgkin lymphoma	ATCC
RPMI-6666	Blood/marrow	Hodgkin disease	ATCC
RPMI-8226	Blood/marrow	Myeloma, plasmacytoma	ATCC
SC-1	Blood/marrow	B-cell lymphoma	ATCC
SR	Blood/marrow	Large B cell lymphoma	ATCC
ST486	Blood/marrow	Burkitt's lymphoma	ATCC
SU-DHL-16	Blood/marrow	Non-Hodgkin lymphoma	DSMZ
SU-DHL-4	Blood/marrow	B-cell lymphoma	DSMZ
SU-DHL-5	Blood/marrow	Non-Hodgkin lymphoma	DSMZ

Cell Line	Tissue	Tumor Type	Cell Source
SU-DHL-6	Blood/marrow	B-cell lymphoma	ATCC
SUP-B15	Blood/marrow	Acute lymphoblastic leukemia	ATCC
TANOUE	Blood/marrow	Acute lymphoblastic leukemia	DSMZ
THP-1	Blood/marrow	Acute myeloid leukemia	ATCC
Toledo	Blood/marrow	Diffuse non-Hodgkin lymphoma	ATCC
U266B1	Blood/marrow	Myeloma	ATCC
A204	Bone/muscle	Rhabdomyosarcoma	ATCC
A-673	Bone/muscle	Rhabdomyosarcoma	ATCC
GCT	Bone/muscle	Fibrous histiocytoma	ATCC
HOS	Bone/muscle	Osteosarcoma	ATCC
HT-1080	Bone/muscle	Fibrosarcoma	ATCC
KHOS-240S	Bone/muscle	Osteosarcoma	ATCC
MG-63	Bone/muscle	Osteosarcoma	ATCC
RD	Bone/muscle	Osteosarcoma	ATCC
RD-ES	Bone/muscle	Ewing's sarcoma	ATCC
Saos-2	Bone/muscle	Osteosarcoma	ATCC
SJRH30	Bone/muscle	Rhabdomyosarcoma	ATCC
SW684	Bone/muscle	Fibrosarcoma	ATCC
SW872	Bone/muscle	Liposarcoma	ATCC
SW982	Bone/muscle	Synovial sarcoma	ATCC
U-2 0S	Bone/muscle	Osteosarcoma	ATCC
TE381.T	Bone/muscle	Rhabdomyosarcoma	ATCC
A172	Brain	Glioblastoma	ATCC
CHP-212	Brain	Neuroblastoma	ATCC
D283-Med	Brain	Medulloblastoma	ATCC
DBTRG-05MG	Brain	Glioblastoma	ATCC

Cell Line	Tissue	Tumor Type	Cell Source
DK-MG	Brain	Glioblastoma	DSMZ
H4	Brain	Neuroglioma	ATCC
M059J	Brain	Glioma	ATCC
M059K	Brain	Glioma	ATCC
MC-IXC	Brain	Neuroblastoma	ATCC
SF268	Brain	Glioblastoma	NCI-60
SF295	Brain	Glioblastoma	NCI-60
SF-539	Brain	Glioblastoma	NCI-60
SK-N-AS	Brain	Neuroblastoma	ATCC
SK-N-BE(2)	Brain	Neuroblastoma	ATCC
SK-N-DZ	Brain	Neuroblastoma	ATCC
SK-N-F1	Brain	Neuroblastoma	ECACC
SNB-19	Brain	Glioblastoma	DSMZ
SW1088	Brain	Glioblastoma	ATCC
SW1783	Brain	Glioblastoma	ATCC
U-251	Brain	Glioblastoma	NCI-60
U-87MG	Brain	Glioblastoma	ATCC
AU565	Breast	Breast carcinoma	ATCC
BT-20	Breast	Breast carcinoma	ATCC
BT474	Breast	Breast carcinoma	ATCC
BT-549	Breast	Breast carcinoma	ATCC
DU4475	Breast	Breast carcinoma	DSMZ
EFM-19	Breast	Breast carcinoma	DSMZ
HCC1143	Breast	Breast carcinoma	ATCC
HCC1569	Breast	Breast carcinoma	ATCC
HCC1599	Breast	Breast carcinoma	ATCC

Cell Line	Tissue	Tumor Type	Cell Source
HCC1937	Breast	Breast carcinoma	ATCC
HCC1954	Breast	breast carcinoma	ATCC
HCC202	Breast	Breast carcinoma	ATCC
HCC2157	Breast	Breast carcinoma	ATCC
HCC2218	Breast	Breast carcinoma	ATCC
HCC38	Breast	Breast carcinoma	ATCC
HS578T	Breast	Breast carcinoma	ATCC
KPL-1	Breast	Breast carcinoma	DSMZ
MDA-MB-157	Breast	Breast medullary carcinoma	ATCC
MDA-MB-231	Breast	Breast carcinoma	ATCC
MDA-MB-361	Breast	Breast carcinoma	ATCC
MDA-MB-436	Breast	Breast carcinoma	ATCC
MDA-MB-453	Breast	Breast carcinoma	ATCC
MDA-MB-468	Breast	Breast carcinoma	ATCC
MT-3	Breast	Breast carcinoma	DSMZ
NCI/ADR RES	Breast	Breast carcinoma	NCI-60
SK-BR-3	Breast	Breast carcinoma	ATCC
T-47Dt	Breast	Breast carcinoma	ATCC
UACC-812	Breast	Breast carcinoma	ATCC
UACC-893	Breast	Breast carcinoma	ATCC
ZR-75-1	Breast	Breast carcinoma	ATCC
C-33 A	Cervix	Cervical carcinoma	ATCC
C-4I	Cervix	Cervical carcinoma	ATCC
C-4II	Cervix	Cervical carcinoma	ATCC
DoTc2-4510	Cervix	Cervical carcinoma	ATCC
HELA	Cervix	Cervical carcinoma	ATCC

Cell Line	Tissue	Tumor Type	Cell Source
COLO-201	Colorectal	Colorectal carcinoma	ATCC
COLO-205	Colorectal	Colorectal adenocarcinoma	ATCC
COLO-320DM	Colorectal	Colorectal adenocarcinoma	ATCC
COLO-320HSR	Colorectal	Colon adenocarcinoma	ATCC
DLD-1	Colorectal	Colon carcinoma	ATCC
HCC-2998	Colorectal	Colorectal carcinoma	NCI-60
HCT-15	Colorectal	Colon adenocarcinoma	ATCC
HCT-8	Colorectal	Ileocecal adenocarcinoma	ATCC
KM12	Colorectal	Colon adenocarcinoma	NCI-60
LoVo	Colorectal	Colon adenocarcinoma	ATCC
LS1034	Colorectal	Colorectal carcinoma	ATCC
LS-174T	Colorectal	Colon adenocarcinoma	ATCC
NCI-H508	Colorectal	Colorectal carcinoma	ATCC
NCI-H630	Colorectal	Rectal carcinoma	ATCC
NCI-H716	Colorectal	Colorectal carcinoma	ATCC
NCI-H747	Colorectal	Colorectal carcinoma	ATCC
RKO	Colorectal	Colon carcinoma	ATCC
RKO-E6	Colorectal	Colon carcinoma	ATCC
SW1116	Colorectal	Colon adenocarcinoma	ATCC
SW1417	Colorectal	Colon adenocarcinoma	ATCC
SW1463	Colorectal	Rectal carcinoma	ATCC
SW403	Colorectal	Colon adenocarcinoma	ATCC
SW48	Colorectal	Colon adenocarcinoma	ATCC
SW480	Colorectal	Colorectal adenocarcinoma	ATCC
SW-620	Colorectal	Colorectal carcinoma	NCI-60
SW837	Colorectal	Rectal carcinoma	ATCC

Cell Line	Tissue	Tumor Type	Cell Source
SW948	Colorectal	Colorectal adenocarcinoma	ATCC
T84	Colorectal	Colorectal adenocarcinoma	ATCC
WIDR	Colorectal	Colorectal adenocarcinoma	ATCC
KYSE-30	Esophageal	Esophageal carcinoma	DSMZ
OE19	Esophageal	Esophageal carcinoma	ECACC
OE33	Esophageal	Esophageal carcinoma	ECACC
CAL-27	Head/neck	Squamous cell carcinoma of tongue	ATCC
Detroit562	Head/neck	Carcinoma of pharynx	ATCC
FaDu	Head/neck	Squamous cell carcinoma of pharynx	ATCC
H157	Head/neck	Buccal mucosa squamous carcinoma	ECACC
RPMI-2650	Head/neck	Nasal squamous cell carcinoma	ATCC
SCC-15	Head/neck	Squamous cell carcinoma of tongue	ATCC
SCC-25	Head/neck	Squamous cell carcinoma of tongue	ATCC
SCC-4	Head/neck	Squamous cell carcinoma of tongue	ATCC
769-P	Kidney	Renal cell adenocarcinoma	ATCC
786-O	Kidney	Renal cell carcinoma	ATCC
A-498	Kidney	Renal cell carcinoma	ATCC
A704	Kidney	Renal cell adenocarcinoma	ATCC
ACHN	Kidney	Renal cell carcinoma	ATCC
Caki-1	Kidney	Clear cell carcinoma	ATCC
G-401	Kidney	Malignant rhabdoid	ATCC
G-402	Kidney	Leiomyoblastoma	ATCC
SN12C	Kidney	Renal cell carcinoma	NCI-60
TK-10	Kidney	Renal cell carcinoma	NCI-60
UO-31	Kidney	Renal cell carcinoma	NCI-60
Hep-3B	Liver	Hepatocellular carcinoma	ATCC

Cell Line	Tissue	Tumor Type	Cell Source
HepG2	Liver	Hepatocellular carcinoma	ATCC
SNU-182	Liver	Hepatocellular carcinoma	ATCC
SNU-387	Liver	Hepatocellular carcinoma	ATCC
SNU-475	Liver	Hepatocellular carcinoma	ATCC
A-427	Lung	Lung carcinoma	DSMZ
A549	Lung	Non-small cell lung carcinoma	DSMZ
Calu-1	Lung	Epidermoid carcinoma of the lung	ATCC
Calu-6	Lung	Adenocarcinoma of lung	ATCC
ChaGo-K-1	Lung	Squamous cell carcinoma of bronchus	ATCC
COR-L105	Lung	Lung adenocarcinoma	ECACC
COR-L279	Lung	Small cell lung cancer	ECACC
COR-L88	Lung	Small cell lung cancer	ECACC
DMS 114	Lung	Small cell lung carcinoma	ATCC
DMS 153	Lung	Small cell lung carcinoma	ATCC
DMS 273	Lung	Small cell lung carcinoma	ECACC
DMS 53	Lung	Small cell lung carcinoma	ATCC
EKVX	Lung	Lung adenocarcinoma	
HOP-62	Lung	Adenocarcinoma	NCI-60
HOP-92	Lung	Large cell carcinoma of lung	NCI-60
NCH-H1651	Lung	Non-small cell lung cancer	ATCC
NCI -H1155	Lung	Non-small cell lung carcinoma	ATCC
NCI-H1048	Lung	Small cell lung carcinoma	ATCC
NCI-H1092	Lung	Small cell lung carcinoma	ATCC
NCI-H1299	Lung	Non-small cell lung carcinoma	ATCC
NCI-H1395	Lung	Lung adenocarcinoma	ATCC
NCI-H1437	Lung	Non-small cell lung carcinoma	ATCC

Cell Line	Tissue	Tumor Type	Cell Source
NCI-H1563	Lung	Lung adenocarcinoma	ATCC
NCI-H1573	Lung	Adenocarcinoma of lung	ATCC
NCI-H1581	Lung	Large cell carcinoma of lung	ATCC
NCI-H1618	Lung	Small cell carcinoma of lung	ATCC
NCI-H1623	Lung	Non-small cell lung carcinoma	ATCC
NCI-H1650	Lung	Squamous cell carcinoma of bronchus	ATCC
NCI-H1666	Lung	Bronchioalveolar adenocarcinoma	ATCC
NCI-H1694	Lung	Squamous cell carcinoma of bronchus	ATCC
NCI-H1703	Lung	Mixed adenosquamous lung carcinoma	ATCC
NCI-H1770	Lung	Large cell neuroendocrine carcinoma	ATCC
NCI-H1793	Lung	Adenocarcinoma of lung	ATCC
NCI-H1838	Lung	Adenocarcinoma of lung	ATCC
NCI-H187	Lung	Small cell lung carcinoma	ATCC
NCI-H1930	Lung	Small cell lung carcinoma	ATCC
NCI-H1975	Lung	Adenocarcinoma of lung	ATCC
NCI-H1993	Lung	Adenocarcinoma of lung	ATCC
NCI-H2009	Lung	Adenocarcinoma of lung	ATCC
NCI-H2030	Lung	Non small cell lung carcinoma	ATCC
NCI-H2052	Lung	Lung mesothelioma	ATCC
NCI-H2081	Lung	Small cell lung carcinoma	ATCC
NCI-H2087	Lung	Lung adenocarcinoma	ATCC
NCI-H2122	Lung	Lung adenocarcinoma	ATCC
NCI-H2126	Lung	Non-small cell lung cancer	ATCC
NCI-H2170	Lung	Lung squamous cell carcinoma	ATCC
NCI-H2171	Lung	Small cell lung carcinoma	ATCC
NCI-H226	Lung	Mesothelioma	ATCC

Cell Line	Tissue	Tumor Type	Cell Source
NCI-H23	Lung	Non-small cell lung carcinoma	ATCC
NCI-H2347	Lung	Non-small cell lung carcinoma	ATCC
NCI-H2405	Lung	Lung adenocarcinoma	ATCC
NCI-H292	Lung	Mucoepidermoid carcinoma	ATCC
NCI-H358	Lung	Bronchioalveolar carcinoma	ATCC
NCI-H441	Lung	Papillary adenocarcinoma	ATCC
NCI-H446	Lung	Small cell lung carcinoma	ATCC
NCI-H460	Lung	Large cell lung carcinoma	ATCC
NCI-H510	Lung	Small cell lung carcinoma	ATCC
NCI-H522	Lung	Small cell lung carcinoma	ATCC
NCI-H526	Lung	Small cell lung carcinoma	ATCC
NCI-H596	Lung	Adenosquamous carcinoma	ATCC
NCI-H650	Lung	Bronchioalveolar adenocarcinoma	ATCC
NCI-H661	Lung	Non-small cell lung carcinoma	ATCC
NCI-H69	Lung	Small cell lung carcinoma	ATCC
NCI-H720	Lung	Atypical carcinoma	ATCC
NCI-H727	Lung	Pulmonary carcinoid	ATCC
NCI-H810	Lung	Large cell carcinoma	ATCC
NCI-H82	Lung	Small cell lung carcinoma	ATCC
NCI-H838	Lung	Non-small cell lung carcinoma	ATCC
SHP-77	Lung	Small cell lung carcinoma	ATCC
SK-MES-1	Lung	Squamous cell lung carcinoma	ATCC
SW1573	Lung	Adenocarcinoma	ATCC
SW900	Lung	Squamous cell carcinoma of lung	ATCC
UMC-11	Lung	Pulmonary carcinoma	ATCC
CaOV3	Ovary	Ovarian carcinoma	ATCC

Cell Line	Tissue	Tumor Type	Cell Source
COLO-704	Ovary	Ovarian carcinoma	DSMZ
OV-90	Ovary	Malignant papillary serous adenocarcinoma	ATCC
OVCAR4	Ovary	Ovarian carcinoma	NCI-60
OVCAR-8	Ovary	Ovarian carcinoma	NCI-60
SKOV3	Ovary	Ovarian carcinoma	NCI-60
AsPC-1	Pancreas	Pancreatic carcinoma	ATCC
BxPC-3	Pancreas	Pancreatic adenocarcinoma	ATCC
Capan-1	Pancreas	Pancreatic adenocarcinoma	ATCC
Capan-2	Pancreas	Pancreatic carcinoma	ATCC
HPAC	Pancreas	Pancreatic carcinoma	ATCC
HPAF-II	Pancreas	Pancreatic adenocarcinoma	ATCC
HuP-T4	Pancreas	Pancreatic adenocarcinoma	ECACC
MiaPaCa	Pancreas	Pancreatic carcinoma	ATCC
Panc02.03	Pancreas	Pancreatic adenocarcinoma	ATCC
Panc08.13	Pancreas	Pancreatic carcinoma	ATCC
PANC-1	Pancreas	Pancreatic epithelioid carcinoma	ATCC
YAPC	Pancreas	Pancreatic carcinoma	DSMZ
PL45	Pancreas	Pancreatic adenocarcinoma	ATCC
BeWo	Placenta	Choriocarcinoma	ATCC
JAR	Placenta	Choriocarcinoma	ATCC
JEG-3	Placenta	Choriocarcinoma	ATCC
22Rv1	Prostate	Prostate carcinoma	ATCC
BM-1604	Prostate	Prostate carcinoma	DSMZ
CA-HPV-10	Prostate	Prostate carcinoma	ATCC
DU145	Prostate	Prostate carcinoma	ATCC
LNCaP	Prostate	Prostate carcinoma	ATCC

Cell Line	Tissue	Tumor Type	Cell Source
PC3	Prostate	Prostate carcinoma	ATCC
RWPE-1	Prostate	Prostate carcinoma	ATCC
Y79	Retina	Retinoblastoma	ATCC
A101D	Skin	Malignant melanoma	ATCC
A375	Skin	Malignant melanoma	ATCC
A375P	Skin	Malignant melanoma	ECACC
A-431	Skin	Epidermoid carcinoma	ATCC
A7	Skin	Malignant melanoma	ATCC
C32TG	Skin	Malignant melanoma	ATCC
CHL-1	Skin	Malign ant melanoma	ATCC
COLO-829	Skin	Malignant melanoma	ATCC
HMCB	Skin	Malignant melanoma	ATCC
M14	Skin	Malignant melanoma	NCI-60
MALME-3M	Skin	Malignant melanoma	ATCC
SH-4	Skin	Malignant melanoma	ATCC
SK-MEL-1	Skin	Malignant melanoma	ATCC
SK-MEL-2	Skin	Malignant melanoma	ATCC
SK-MEL-28	Skin	Malignant melanoma	ATCC
SK-MEL-3	Skin	Malignant melanoma	ATCC
SK-MEL-5	Skin	Malignant melanoma	ATCC
UACC-62	Skin	Malignant melanoma	NCI-60
WM-115	Skin	Malignant melanoma	ATCC
LOXIMVI	Skin	Malignant melanoma	NCI-60
AGS	Stomach	Adenocarcinoma of the stomach	ATCC
HS746T	Stomach	Adenocarcinoma of the stomach	ATCC
KATO_III	Stomach	Carcinoma of the stomach	ATCC

Cell Line	Tissue	Tumor Type	Cell Source
NCI-N87	Stomach	Carcinoma of the stomach	ATCC
SNU-1	Stomach	Carcinoma of the stomach	ATCC
SNU-16	Stomach	Carcinoma of the stomach	ATCC
SNU-5	Stomach	Carcinoma of the stomach	ATCC
BHT-101	Thyroid	Thyroid carcinoma	DSMZ
CAL-62	Thyroid	Thyroid carcinoma	DSMZ
CGTH-W-1	Thyroid	Thyroid carcinoma	DSMZ
SW579	Thyroid	Thyroid carcinoma	ATCC
TT	Thyroid	Thyroid carcinoma	ATCC
HEC-1-A	Uterus	Uterine carcinoma	ATCC
HEC-1-B	Uterus	Uterine carcinoma	ATCC
KLE	Uterus	Uterine carcinoma	ATCC
RL95-2	Uterus	Carcinoma of endometrium	ATCC
SK-UT-1	Uterus	Uterine leiomyosarcoma	ATCC
AN3CA	Uterus	Uterus carcinoma	ATCC
SK-LMS-1	Vulva	Vulva leiomyosarcoma	ATCC
SW954	Vulva	Squamous cell carcinoma	ATCC
SW962	Vulva	Carcinoma	ATCC

ATCC, American Type Culture Collection; DSMZ, German Collection of Microorganisms and Cell Cultures; NCI, National Cancer Institute; ECACC, European Collection of Cell Cultures.
